# Cyclic helix B peptide inhibits ischemia reperfusion-induced renal fibrosis via the PI3K/Akt/FoxO3a pathway

**DOI:** 10.1186/s12967-015-0699-2

**Published:** 2015-11-10

**Authors:** Cheng Yang, Ye Cao, Yi Zhang, Long Li, Ming Xu, Yaqiu Long, Ruiming Rong, Tongyu Zhu

**Affiliations:** Department of Urology, Zhongshan Hospital, Fudan University, Shanghai, 200032 China; Shanghai Key Laboratory of Organ Transplantation, 180 Fenglin Road, Shanghai, 200032 China; Department of Chinese Traditional Medicine, Shanghai University of Chinese Traditional Medicine, Shanghai, 201203 China; The Faculty of Life Science and Computing, London Metropolitan University, London, N7 8DB UK; CAS Key Laboratory of Receptor Research, Shanghai Institute of Materia Medica, Chinese Academy of Sciences, Shanghai, 201203 China; Department of Transfusion, Zhongshan Hospital, Fudan University, Shanghai, 200032 China; Qingpu Branch Zhongshan Hospital, Fudan University, 1158 Gongyuan Road East, Shanghai, 201700 China; Department of Plastic Surgery, Zhongshan Hospital, Fudan University, Shanghai, 200032 China

**Keywords:** Cyclic helix B peptide, Renal ischemia reperfusion injury, Fibrosis, Akt, FoxO3

## Abstract

Renal fibrosis is a main cause of end-stage renal disease. Clinically, there is no beneficial treatment that can effectively reverse the progressive loss of renal function. We recently synthesized a novel proteolysis-resistant cyclic helix B peptide (CHBP) that exhibits promising renoprotective effects. In this study, we evaluated the effect of CHBP on renal fibrosis in an in vivo ischemia reperfusion injury (IRI) model and in vitro TGF-β-stimulated tubular epithelial cells (TCMK-1 and HK-2) model. In the IRI in vivo model, mice were randomly divided into sham (sham operation), IR and IR + CHBP groups (n = 6). CHBP (8 nmol/kg) was administered intraperitoneally at the onset of reperfusion, and renal fibrosis was evaluated at 12 weeks post-reperfusion. Our results showed that CHBP markedly attenuated the IRI-induced deposition of collagen I and vimentin. In the in vitro model, CHBP reversed the TGF-β-induced down-regulation of E-cadherin and up-regulation of α-SMA and vimentin. Furthermore, CHBP inhibited the phosphorylation of Akt and Forkhead box O 3a (FoxO3a), whose anti-fibrotic effect could be reversed by the 3-phosphoinositide-dependent kinase-1 (PI3K) inhibitor wortmannin as well as FoxO3a siRNA. These findings demonstrate that CHBP attenuates renal fibrosis and the epithelial-mesenchymal transition of tubular cells, possibly through suppression of the PI3K/Akt pathway and thereby the inhibition FoxO3a activity.

**Electronic supplementary material:**

The online version of this article (doi:10.1186/s12967-015-0699-2) contains supplementary material, which is available to authorized users.

## Background

Acute kidney injury (AKI) is a common clinical syndrome that increases the risk of adverse outcomes. AKI affects >5 % of all inpatients and is especially common in critically ill patients. More than 36 % of patients experience AKI 1 day after admission to an intensive-care unit under a sepsis condition, and the prevalence increases to >60 % while in the intensive-care unit [1]. AKI significantly increases the risk of chronic renal disease, end-stage renal disease, and death, presenting a major burden to patients and the health care system [2]. Renal ischemia reperfusion injury (IRI) is one of the major causes of AKI [3].

Studies have shown that AKI results in incomplete tubular repair, which is characterized by failed differentiation and persistently high signaling activity. IRI of the kidney is a well-established cause of renal fibrosis [4, 5]. Factors such as sustained innate immune cell activation, endothelial cell dysfunction, hypoxia, and chronic microvascular injury have all been implicated in the maladaptive response that results in fibrogenesis and progression to chronic kidney disease [6]. The epithelial-mesenchymal transition (EMT) plays a critical role in the pathogenesis of fibrosis, in which renal tubular epithelial cells (TECs) under pathological conditions can phenotypically convert to a fibroblast-like morphology in the tubulointerstitium [7, 8].

Forkhead box O (FoxO) transcription factors are the human homologues of *C. elegans* transcription factor DAF-16 and share a highly conserved 110-amino acid DNA-binding domain termed the forkhead box or winged-helix domain [9]. Four principal members of the mammalian FoxO subfamily, FoxO1, FoxO3a, FoxO4 and FoxO6, have been described [10]. Among these, FoxO3a has been extensively studied as a crucial protein involved in the regulation of several essential cellular functions. The potency of FoxO3a is carefully regulated by phosphorylation. Activated 3-phosphoinositide-dependent kinase-1 (PI3K) activates serine/threonine protein kinase B (Akt) by recruiting and phosphorylating its lipid substrates [11]. Akt then phosphorylates and inactivates FoxO3a, which results in the retention of FoxO3a in the cytoplasm and subsequent inhibition of target gene transcription. By contrast, FoxO3a dephosphorylation results in its nuclear translocation and activation [11].

Recently, we used a novel cyclization strategy to synthesize thioether cyclic helix B peptide (CHBP), which showed significantly improved metabolic stability and renoprotective effects [12]. In our previous studies in murine IRI models, CHBP exhibited a strong renoprotection effect against IRI in terms of reduced inflammation and apoptosis [12]. In porcine models, CHBP administered using preservation solution and autologous blood perfusate provided significant protection against kidney IRI by increasing renal blood flow and oxygenation and reducing apoptosis, inflammation, as well as tissue structural damage [13]. Based on these results, we hypothesized that CHBP might attenuate IRI-induced renal fibrosis.

In the present study, we investigated the anti-fibrotic potential of CHBP in a murine renal IRI-induced fibrosis model and in cultured proximal tubular epithelial cells (TECs). We also examined the molecular mechanisms of CHBP-mediated renoprotection against fibrosis.

## Methods

### Renal IRI in vivo model

Male BALB/c mice (20–25 g) were obtained from Shanghai Slac Lab Animal, Co., Ltd., and bred in an SPF-grade experimental animal room. The renal IRI model was established as previously described [12, 14]. Each mouse was anesthetized intraperitoneally with pentobarbital at 0.1 g/kg. The core body temperature was maintained at 37 °C using a homeothermic pad during the entire procedure. The abdominal cavity was exposed by a midline incision. Both kidneys were exposed, and the renal pedicles were carefully isolated. Bilateral renal occlusion was performed for 30 min using non-traumatic vascular clamps. Occlusion was confirmed by monitoring the transformation in renal tissue color from red to navy blue. After the clamps were removed, the kidneys were observed for an additional 5 min to ensure that the color returned, which indicated blood reperfusion. Subsequently, 1 ml of saline solution at 37 °C was applied to the abdomen, and the incision was sutured in two layers. For the sham group, both kidneys were exposed, and the renal pedicles were carefully isolated without occlusion. All animal experiments were performed according to the guidelines of the Care and Use of Laboratory Animals of the Laboratory Animal Ethical Commission of Fudan University with good animal surgical research practices and was approved by the Animal Ethical Committee of Zhongshan Hospital, Fudan University.

Immediately after the mice were sutured, they were randomly divided into three groups (n = 6) according to the different treatments: (1) Sham group (without renal pedicles occlusion); (2) IR group (with renal pedicles occlusion); (3) CHBP group (with renal pedicles occlusion and CHBP administration). The IR group received PBS and the CHBP group received PBS containing 8 nmol/kg CHBP by intraperitoneal injection at the onset of reperfusion. Animals were ethically sacrificed at 12 weeks post-reperfusion.

### TECs EMT in vitro model

TCMK-1 and HK-2 cells were purchased from the American Type Culture Collection (ATCC) and cultured in DMEM/F12 medium containing 10 % FBS. Cells were grown in a humidified incubator at 37 °C with 5 % CO_2_ and 95 % air. TCMK-1 and HK-2 cells were seeded into 6-well plates (1 × 10^5^ cells/well) and cultured for 24 h. In the initial in vitro experiment, cells were divided into 4 groups: a control (no TGF-β added) and three groups receiving TGF-β (2.5, 5 or 10 ng/ml). Cells were harvested and processed for western blot analysis at 72 h after the addition of TGF-β to the appropriate wells. In the second experiment, TCMK-1 and HK-2 cells were divided into 5 groups: a control group, a TGF-β (5 ng/ml) group, and three TGF-β + CHBP (0.1, 1 or 10 μM) groups. 72 h after the addition of TGF-β and/or CHBP to the wells, cells were harvested and processed for western blot analysis. In the third experiment, all wells received the same levels of TGF-β (5 ng/ml) and CHBP (10 μM). The wells (HK-2 cells only) were divided into 7 groups: (1) normal (no TGF-β); (2) TGF-β; (3) TGF-β + CHBP; (4) TGF-β + CHBP + wortmannin (0.1 μM); (5) TGF-β + CHBP + FoxO3a siRNA (0.1 μM); (6) TGF-β + CHBP + control siRNA (0.1 μM); and (7) TGF-β + CHBP + wortmannin (0.1 μM) + FoxO3a siRNA (0.1 μM). In each experiment, cells were cultured for 72 h and then harvested and processed for western blot analysis. The efficacy of FoxO3a siRNA (Additional file 1: Figure S1a, b) and wortmannin was tested (Additional file 1: Figure S1c). All experiments were performed in triplicate.

### Histological assessment

Hematoxylin and eosin (H&E) staining was performed to assess histological injury. The tissue sections were blind-labeled and reviewed by two renal pathologists. Renal damage was graded based on the percentage of damaged tubules in the sample: 0 = normal kidney (no damage); 1 = minimal damage (<25 % damage); 2 = mild damage (25–50 % damage); 3 = moderate damage (50–75 % damage); and 4 = severe damage (>75 % damage) similar with previous descriptions [3]. Injury included cell vacuolization, cell necrosis and interstitial infiltration. Scores of 1 or 2 represent mild injury, and scores of 3 or 4 and 5 or 6 represent moderate and severe injuries, respectively.

### Extracellular matrix deposition assay

The deposition of extracellular matrix was evaluated using Masson trichrome and Sirius red (collagen-specific dye) staining as previously described [5]. Image-Pro Plus 6.0 software was used to quantify the level of fibrosis.

### Immunohistochemistry

Immunohistochemical staining of vimentin and collagen I (Abcam, Cambridge, UK) was performed on frozen kidney sections using a DAKO ChemMate EnVision Detection Kit (DAKO, Carpinteria, CA, USA) as previously described [15–17].

### Western blot analysis

Membrane proteins were separated on SDS–polyacrylamide gels and transferred to polyvinylidene difluoride membranes. The membranes were blocked with 5 % milk and incubated overnight with anti-E-cadherin, anti-α-SMA, anti-vimentin, anti-PI3K p85, anti-p-Akt Ser473, anti-p-Akt Th308, anti-Akt, anti-p-FoxO3a Ser253, anti-p-FoxO3a Ser318/321, anti-FoxO3a (Cell Signaling, Beverly, MA, USA, 1:1000), or anti-tubulin (Abcam, Cambridge, UK, 1:10,000) antibodies. The results were analyzed as previously described [15].

### Statistical analysis

Data are presented as the mean ± standard deviation. Statistical analysis (SPSS 18.0 software, SPSS Inc., Armonk, NY, USA) was performed using the two-tailed independent Student’s *t* test after a demonstration of homogeneity of variance with the F test or one-way ANOVA for more than two groups. The Scheffe test was used for post hoc analysis. Statistical significance was set as *p* < 0.05.

## Results

### CHBP attenuates renal IRI-induced tissue injury, fibrosis and collagen deposition

**Fig. 1:**
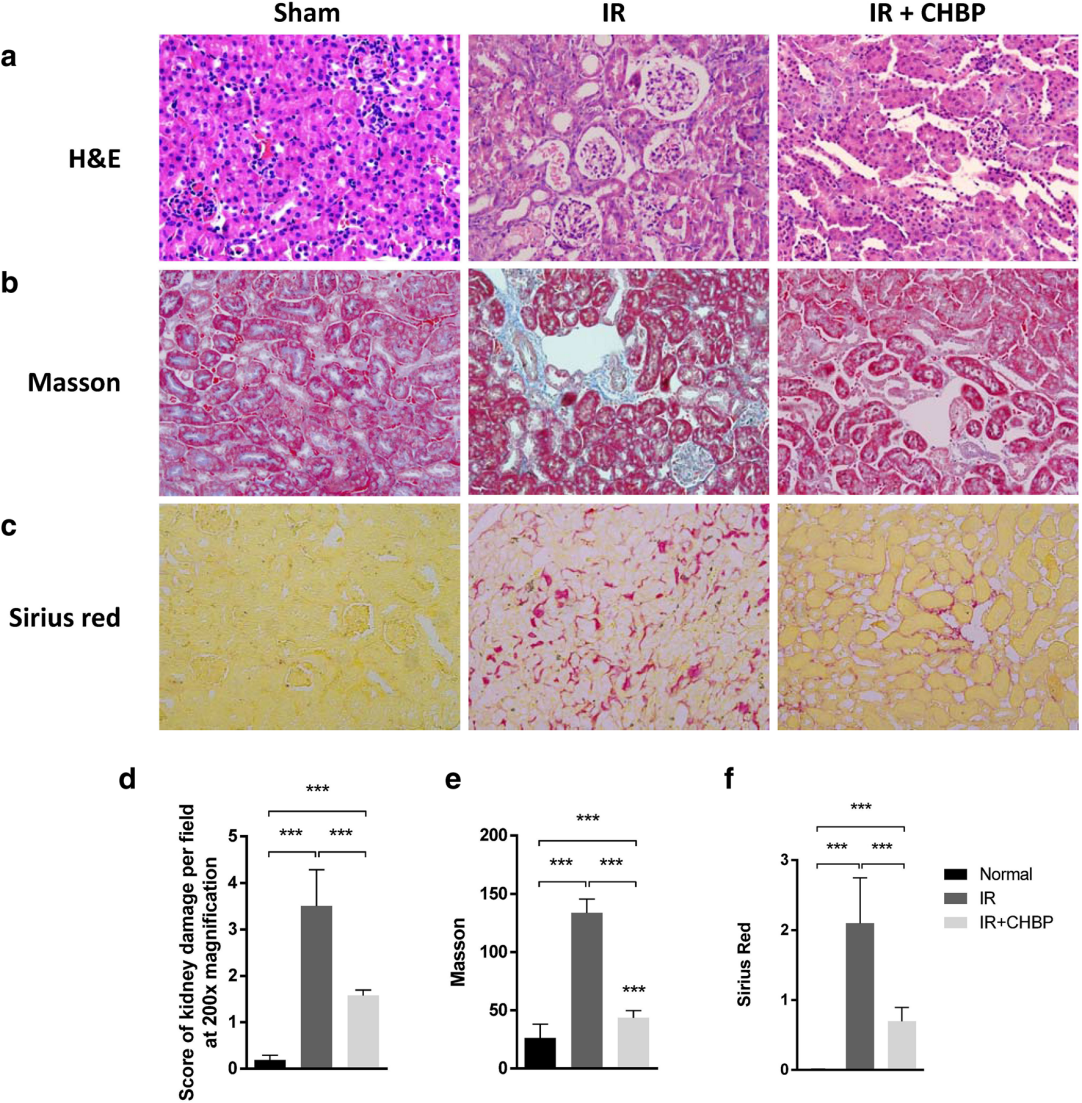
CHBP attenuated IRI-induced renal tissue injury, fibrosis and collagen deposition. **a** H&E, **b** Masson trichrome and **c** Sirius red staining were performed on kidney sections. 12 weeks after reperfusion. **d**, **e**, **f** Semi-quantitative analysis for H&E, Masson trichrome and Sirius red staining, respectively. Data are presented as the mean ± SD, n = 6

To evaluate the renoprotective effects of CHBP in the murine renal IRI model, we performed H&E staining of the kidneys in each group at 12 weeks post-reperfusion (Fig. 1a). Semi-quantitative analysis using a histological scoring system revealed that the kidney tissue in the CHBP-treated group was well protected, showing mild interstitial edema and cellular infiltration. Severe interstitial edema and cellular infiltration with tubular epithelial cell vacuolation were found in the IR group (Fig. 1d).

Masson trichrome staining was used to demonstrate extracellular matrix (ECM) deposition within the tubulointerstitium (Fig. 1b), and ECM deposition was evaluated by Sirius red staining, which intercalates tertiary grooves in the structures of collagen I and III (Fig. 1c). Both stains indicated that renal fibrosis and collagen deposition were elevated in the IR group but markedly reduced by CHBP treatment (Fig. 1e, f).

### CHBP decreases vimentin and collagen I expression in IRI kidneys

**Fig. 2:**
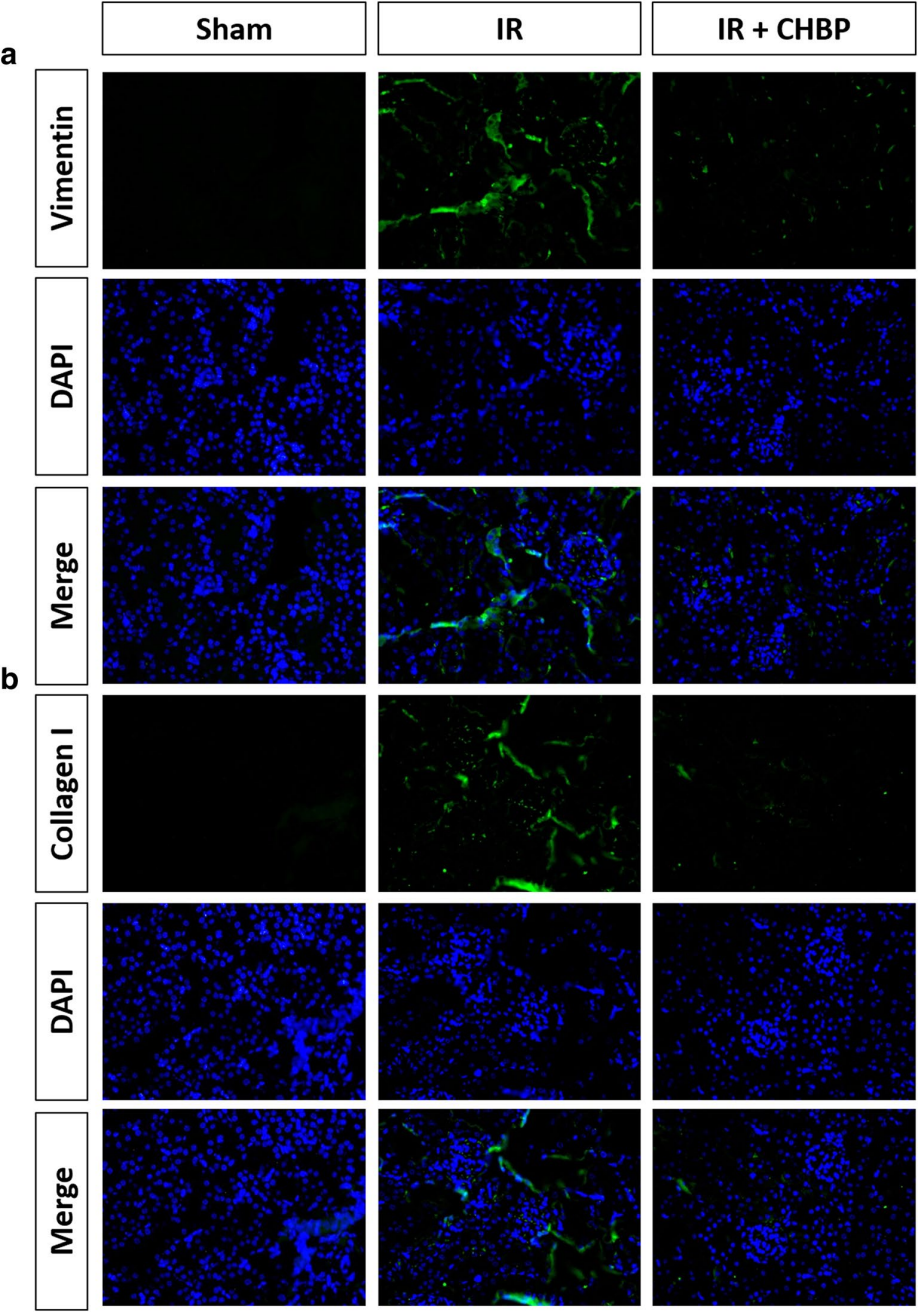
CHBP decreased IRI-induced vimentin and collagen I expression. Immunofluorescence staining was performed for **a** vimentin and **b** collagen I

Next, we determined the effect of CHBP on the expression of myofibroblast activation marker vimentin. Immunofluorescence (IF) analysis showed that the expression of vimentin was increased in the IR group compared with the sham group, suggesting that myofibroblast activation was stimulated by IRI. However, treatment with CHBP significantly inhibited the IRI-induced up-regulation of vimentin (Fig. 2a). Collagen I is the most abundant collagen of the human body. It is the end product in tissue repair, and is present in scar tissue. IF staining indicated that CHBP decreased the expression of collagen I (Fig. 2b) in kidneys. These results demonstrate that CHBP reduces myofibroblast activation and fibrosis.

### TGF-β stimulates EMT in murine and human TECs

**Fig. 3:**
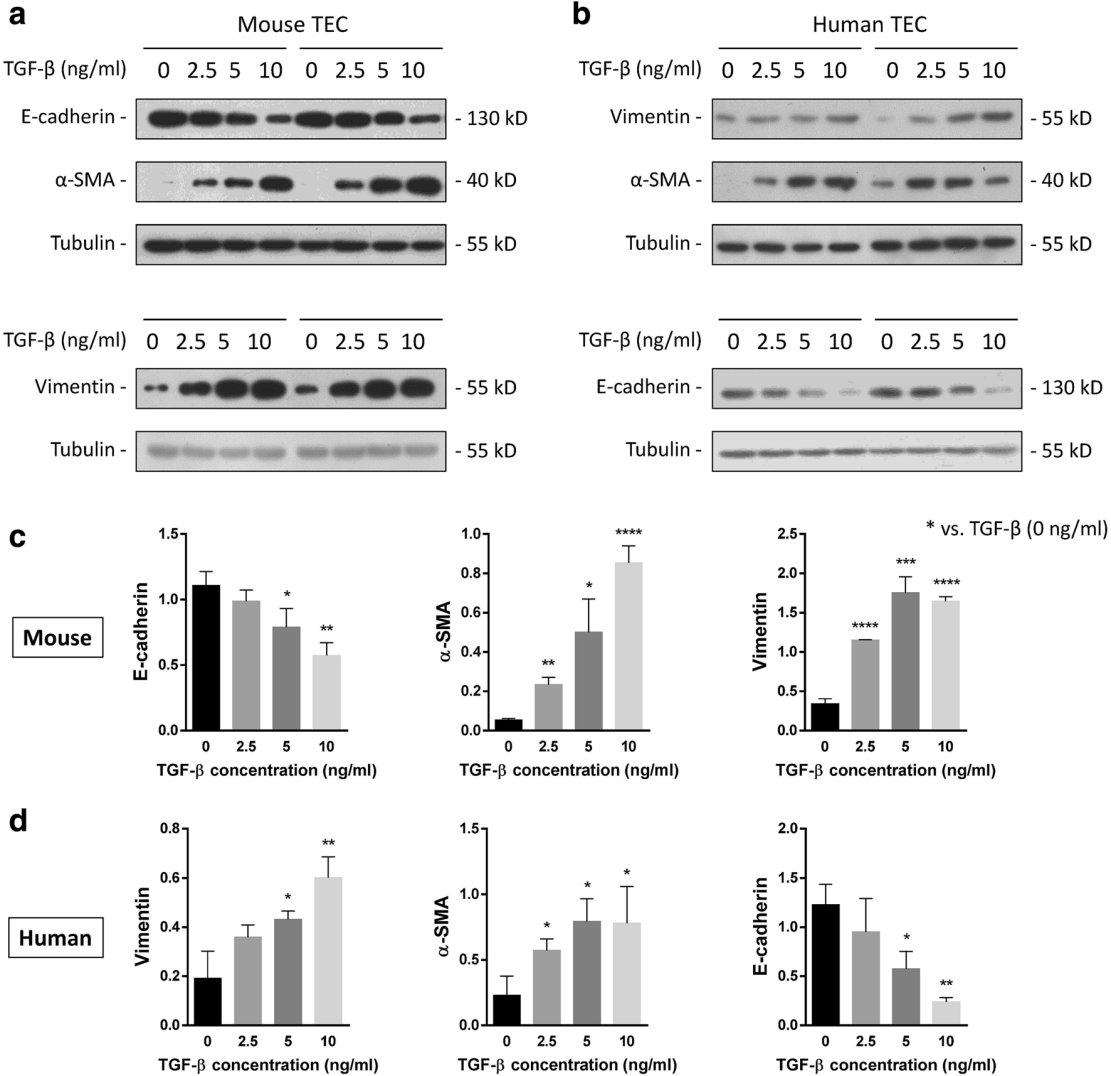
TGF-β induced EMT in murine and human TECs. TGF-β stimulation was performed in murine TCMK-1 and human HK-2 cells. After 72 h, the levels of E-cadherin, vimentin and α-SMA in **a** TCMK-1 and **b** human HK-2 cells with varied levels of TGF-β (0, 2.5, 5 and 10 ng/ml) were determined by western blotting. **c**, **d** Quantitative results presented as the mean ± SD of the optical density of each band, n = 6. *p < 0.05 compared with the TGF-β control group (0 ng/ml)

TGF-β enhances ECM gene regulation and stimulates ECM protein accumulation in renal cells by increasing the expression of protease inhibitors [18]. To determine the optimal concentration of TGF-β for use in culture, we first tested a range of concentrations (0, 2.5, 5 and 10 ng/ml for 72 h) in murine (Fig. 3a) and human (Fig. 3b) TECs. Western blotting analysis revealed that TGF-β significantly increased the expression of a-SMA and vimentin while concomitantly inhibiting E-cadherin expression compared with the control groups in both murine (Fig. 3c) and human (Fig. 3d) TECs. The pro-EMT effects of TGF-β were dependent on the concentration, and 5 ng/ml was identified as the optimal concentration.

### CHBP inhibits TGF-β-stimulated EMT in murine and human TECs

**Fig. 4:**
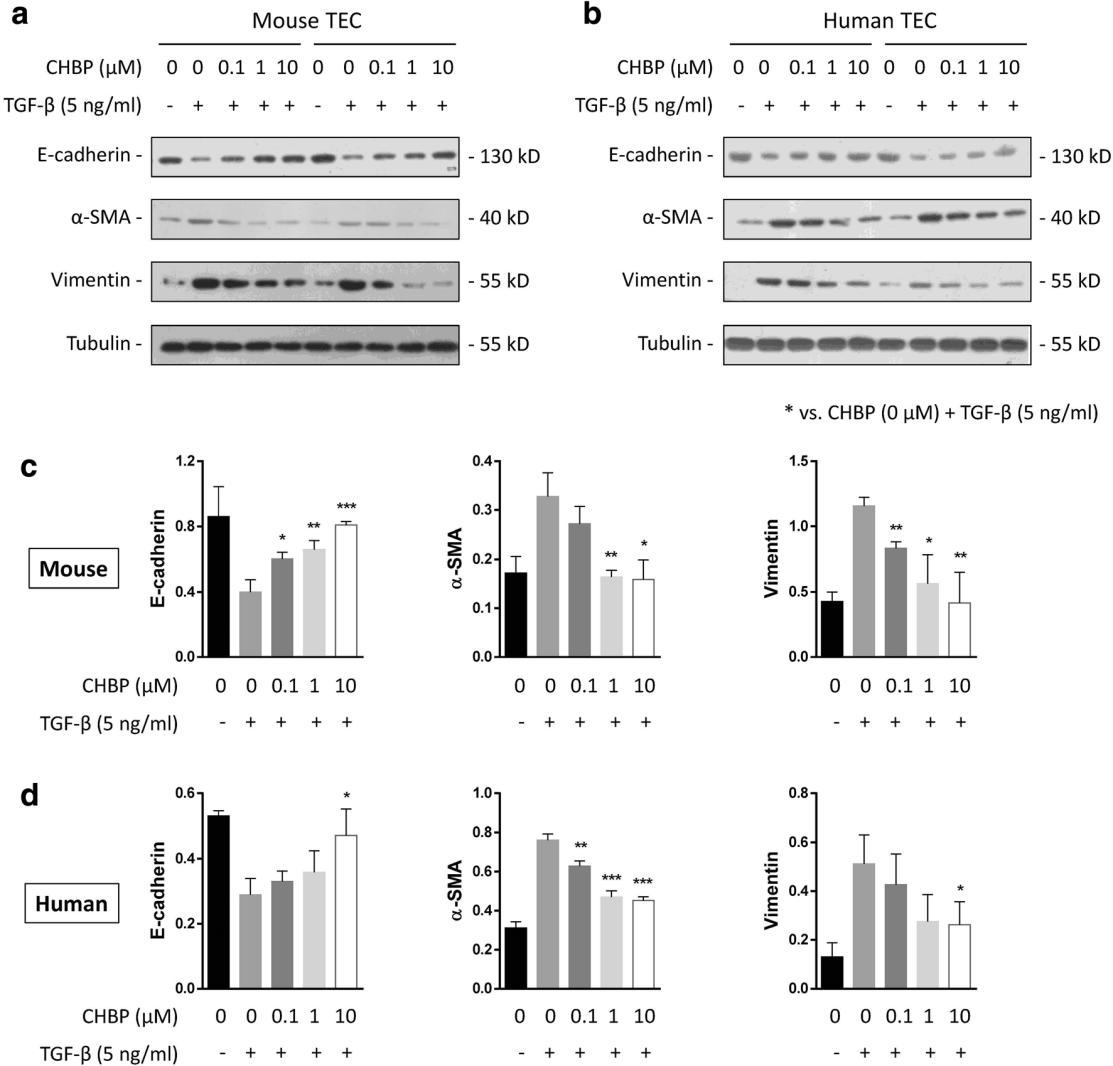
CHBP inhibited TGF-β-induced EMT in murine and human TECs. TGF-β (5 ng/ml) and CHBP (0, 0.1, 1 and 10 μM) were added to murine TCMK-1 and human HK-2 cells. After 72 h, E-cadherin, vimentin and α-SMA levels in **a** TCMK-1 cells and **b** HK-2 cells were determined by western blotting. **c**, **d** Quantitative results are presented as the mean ± SD of the optical density of each band (n = 6). *p < 0.05, **p < 0.01, ***p < 0.001, ****p < 0.0001 compared with the CHBP (0 μM) + TGF-β (5 ng/ml) group

The expression levels of E-cadherin, vimentin and a-SMA, all well-known markers of EMT, were analyzed in murine (Fig. 4a) and human (Fig. 4b) TECs using 5 ng/ml TGF-β. Western blotting analysis revealed that CHBP significantly inhibited the expression of a-SMA and vimentin and enhanced the expression of E-cadherin compared with the control groups in both murine (Fig. 4c) and human (Fig. 4d) TECs. The anti-EMT effects of CHBP were concentration-dependent.

### CHBP blocks PI3K/Akt/FoxO3a signaling

**Fig. 5:**
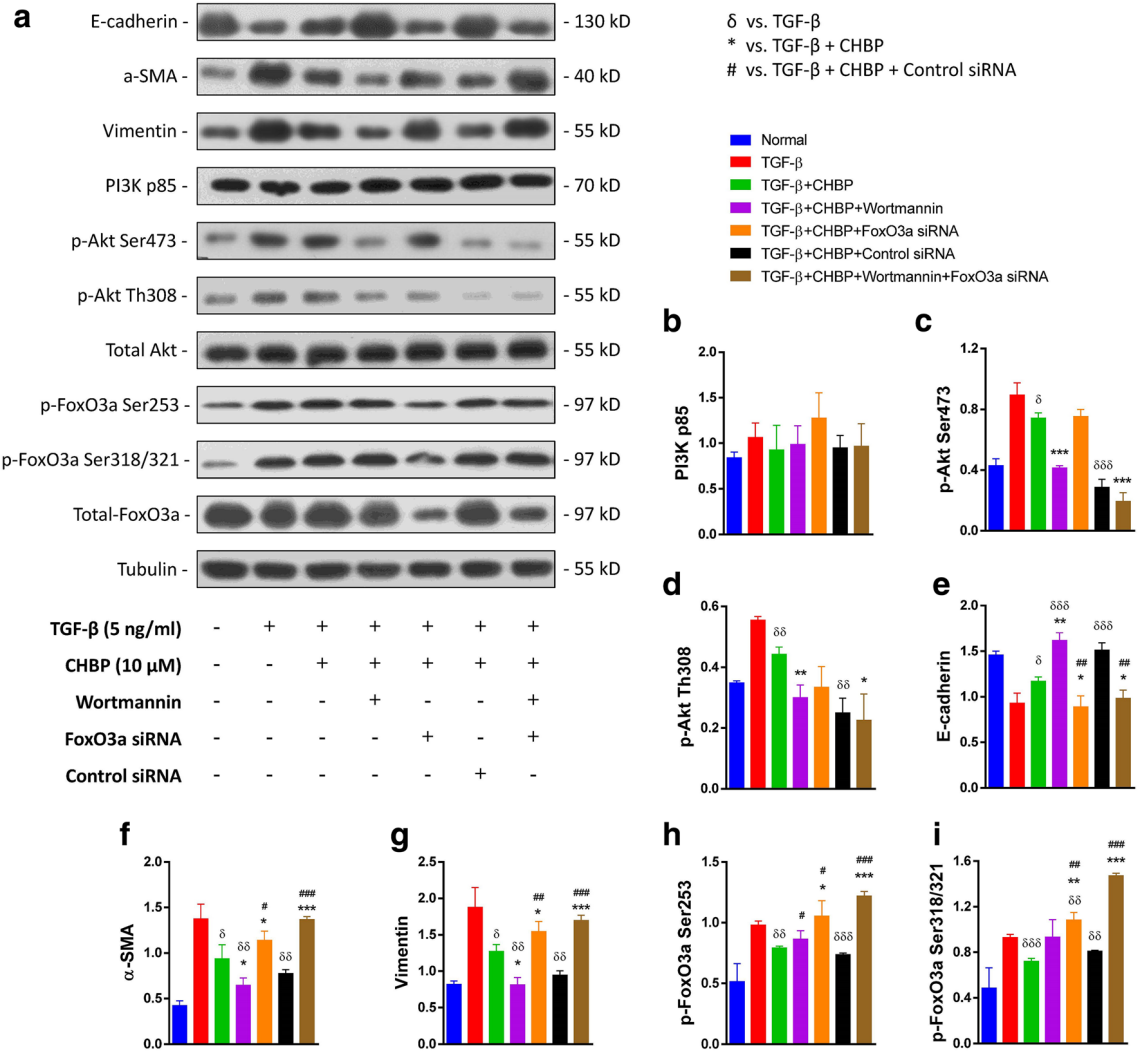
CHBP blocked PI3 K/Akt/FoxO3a signaling. **a** Western blotting analysis of the PI3K/Akt/FoxO3a pathway. Quantitative results for **b** PI3K p85, **c** p-Akt Ser473, **d** p-Akt Th308, **e** E-cadherin, **f** α-SMA, **g** vimentin, **h** p-FoxO3a Ser253, and **i** p-FoxO3a Ser318/321 presented as the mean ± SD of the optical density of each band (n = 6). ^δ^ *p* < 0.05, ^δδ^ *p* < 0.01, ^δδδ^ *p* < 0.001 compared with the TGF-β group. *p < 0.05, **p < 0.01, ***p < 0.001 compared with the TGF-β + CHBP group. ^#^ *p* < 0.05, ^##^ *p* < 0.01, ^###^ *p* < 0.001 compared with the TGF-β + CHBP + control siRNA group

To further investigate the potential mechanism of CHBP-mediated anti-EMT gene regulation in TECs, we performed western blotting analysis targeting the PI3K/Akt/FoxO3a pathway (Fig. 5a). CHBP significantly decreased the levels of phosphorylated Akt (p-Akt) and phosphorylated FoxO3a (p-FoxO3a) (Fig. 5c, d, h, i); these changes were accompanied by an increase in E-cadherin and decrease in α-SMA and vimentin (Fig. 5e–g). In combination with the PI3K inhibitor wortmannin, CHBP further up-regulated or down-regulated the EMT-associated markers, suggesting that the PI3K/Akt pathway is involved in the anti-EMT mechanism of CHBP. In addition, CHBP caused a marked reduction in the phosphorylation of FoxO3a. The anti-EMT effects of CHBP were reversed by FoxO3a siRNA, as evidenced by decreased E-cadherin and increased α-SMA and vimentin levels (Fig. 5e–g), suggesting that the modulation of FoxO3a phosphorylation by CHBP plays a key role in this mechanism. Additionally, FoxO3a siRNA blocked the anti-EMT effects induced by the combination of CHBP and wortmannin.

## Discussion

In this study, we demonstrated for the first time that the novel peptide CHBP ameliorates IRI-induced renal fibrosis by inhibiting the EMT of TECs. The anti-EMT effect of CHBP is mediated by the PI3K/Akt/FoxO3a signaling pathway.

Renal fibrosis is the final consequence of chronic kidney disease, leading to end-stage renal disease via the destruction of the kidney parenchyma and a progression of renal malfunctions [19]. The abnormal ECM that occurs during renal fibrosis results from an overexpression of normal extracellular matrix components and a pathological accumulation of extracellular matrix components such as collagen I. The latter proteins are involved in the renal scarring process and are irreversibly deposited in renal fibrotic tissues. In the present study, we demonstrated that CHBP inhibits pathological extracellular matrix deposition in vivo.

The mechanism of renal fibrosis is associated with TEC transition to a mesenchymal cell phenotype via a process known as EMT [20]. TGF-β is a well-known central mediator in this process [18]. In our study, both murine and human TECs showed dose-dependent up-regulation of EMT-associated proteins in response to TGF-β stimulation. CHBP blocked this effect in both murine and human TECs, and the anti-fibrotic effect of CHBP was dependent on the dosage.

Akt is normally maintained in an inactivated state and is activated by phosphorylation at two sites (Th308 and Ser473) via a PI3K-dependent process. Activated p-Akt regulates many cellular processes, such as proliferation, survival, growth, metabolism and angiogenesis, via serine and/or threonine phosphorylation of downstream substrates [21]. Growing evidence suggests that p-Akt plays a role in renal fibrosis and kidney dysfunction. For example, the PI3 K inhibitor LY294002 was observed to reduce proliferation and ECM synthesis in fibroblasts obtained from rat kidney tissue after unilateral ureteral obstruction [22]. In addition, pharmacological down-regulation of the PI3K/Akt signaling pathway not only reduced the number of pro-fibrotic interstitial cells but also suppressed EMT-associated α-SMA expression [23]. In a recent kidney transplant model, increased expression of p-Akt was found to correlate with allograft interstitial fibrosis [24]. In our study, we found that the levels of p-Akt in murine and human TECs were significantly increased by TGF-β and decreased by CHBP treatment. Reduced p-Akt levels in the CHBP-treated TECs correlated with a lower level of EMT, as indicated by higher levels E-cadherin and lower levels of α-SMA and vimentin. In the TECs treated with a combination of CHBP and wortmannin (a PI3K inhibitor), EMT was further inhibited owing to stronger inhibition of Akt signaling.

**Fig. 6:**
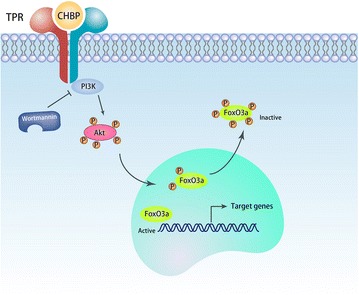
Signaling pathways involved in the inhibition of EMT by CHBP. CHBP stimulates the PI3 K/Akt signaling pathway to phosphorylate FoxO3a, leading to cytoplasmic sequestration of the transcription factor and inactivation of transcriptional activity. The absence of FoxO3a transcriptional activity results in decreased EMT-associated gene expression. Wortmannin can also inhibit PI3 K activity

Phosphorylation of FoxO3a by Akt retains FoxO3a in the cytoplasm, thereby inhibiting its activity [11]. Accordingly, CHBP was shown to decrease the levels of p-FoxO3a in TGF-β-treated TECs, reflecting enhanced activity of FoxO3a in the nucleus. Importantly, FoxO3a siRNA abrogated the anti-fibrotic effect of CHBP, whereas the negative control siRNA had no effect, indicating that the CHBP-mediated reduction in EMT is dependent on Akt and FoxO3a. Additionally, FoxO3a siRNA abrogated the strong anti-fibrotic effects of CHBP and wortmannin in HK-2 cells, suggesting that the pro-fibrotic effect of Akt signaling is dependent on FoxO3a (Fig. 6).

With continued advance in biochemistry and biotechnology, the use of therapeutic peptides has become increasingly effective and popular. CHBP is a conformationally constrained cyclized peptide derived from helix B surface peptide (HBSP). HBSP is a linear peptide derived from erythropoietin and has a 9-min plasma half-life, which restricts its clinical application in vivo [12]. Recently, van Rijt reported that HBSP attenuated renal fibrosis in a porcine kidney model with 45 min of ischemia followed by 7 days of reperfusion [25]. Therefore, as a conformationally constrained cyclized peptide with improved stability and renoprotective properties [12], CHBP may become a promising new drug for the treatment of renal fibrosis.

In conclusion, our study demonstrated that CHBP attenuates renal injury in an IRI murine model by preventing ECM deposition and fibrosis. CHBP also inhibits TGF-β-induced ECM protein expression and EMT in both murine and human TECs. The mechanism of the anti-fibrotic effect of CHBP may involve the suppression of the PI3K/Akt pathway and subsequent inhibition of FoxO3a activity.

## Additional file

10.1186/s12967-015-0699-2 Validation of FoxO3a siRNA and Wortmannin. The p-FoxO3a proteins (A) and mRNA (B) expression of HK-2 cells were significantly down-regulated by FoxO3a shRNA treatment. The expression of p-Akt was also significantly reduced by Wortmannin in the HK-2 cells (C).

## Abbreviations

AKI: acute kidney injury
EMT: epithelial-mesenchymal transition
TECs: renal tubular epithelial cells
Akt: serine/threonine protein kinase B
CHBP: cyclic helix B peptide
IRI: ischemia reperfusion injury
FoxO3a: Forkhead box O 3a
PI3K: 3-phosphoinositide-dependent kinase-1
H&E: hematoxylin and eosin
IF: immunofluorescence
HBSP: helix B surface peptide

## References

[1] Lameire NH, Bagga A, Cruz D, De Maeseneer J, Endre Z, Kellum JA, Liu KD, Mehta RL, Pannu N, Van Biesen W, Vanholder R (2013). Acute kidney injury: an increasing global concern. Lancet.

[2] Kinsey GR, Sharma R, Okusa MD (2013). Regulatory T cells in AKI. J Am Soc Nephrol.

[3] Hu L, Yang C, Zhao T, Xu M, Tang Q, Yang B, Rong R, Zhu T (2012). Erythropoietin ameliorates renal ischemia and reperfusion injury via inhibiting tubulointerstitial inflammation. J Surg Res.

[4] Bonventre JV, Yang L (2011). Cellular pathophysiology of ischemic acute kidney injury. J Clin Invest.

[5] Yang B, Jain S, Pawluczyk IZ, Imtiaz S, Bowley L, Ashra SY, Nicholson ML (2005). Inflammation and caspase activation in long-term renal ischemia/reperfusion injury and immunosuppression in rats. Kidney Int.

[6] Danobeitia JS, Djamali A, Fernandez LA (2014). The role of complement in the pathogenesis of renal ischemia-reperfusion injury and fibrosis. Fibrogenesis Tissue Repair.

[7] Liu Y (2010). New insights into epithelial-mesenchymal transition in kidney fibrosis. J Am Soc Nephrol.

[8] Masola V, Zaza G, Onisto M, Lupo A, Gambaro G (2015). Impact of heparanase on renal fibrosis. J Transl Med.

[9] Clark KL, Halay ED, Lai E, Burley SK (1993). Co-crystal structure of the HNF-3/fork head DNA-recognition motif resembles histone H5. Nature.

[10] Huang H, Tindall DJ (2007). Dynamic FoxO transcription factors. J Cell Sci.

[11] Nho RS, Hergert P (2014). FoxO3a and disease progression. World J Biol Chem.

[12] Yang C, Xu Z, Zhao Z, Li L, Zhao T, Peng D, Xu M, Rong R, Long YQ, Zhu T (2014). A novel proteolysis-resistant cyclic helix B peptide ameliorates kidney ischemia reperfusion injury. Biochim Biophys Acta.

[13] Yang C, Hosgood SA, Meeta P, Long Y, Zhu T, Nicholson ML, Yang B (2015). Cyclic helix B peptide in preservation solution and autologous blood perfusate ameliorates ischemia-reperfusion injury in isolated porcine kidneys. Transplantation Direct.

[14] Yang C, Zhao T, Lin M, Zhao Z, Hu L, Jia Y, Xue Y, Xu M, Tang Q, Yang B (2013). Helix B surface peptide administered after insult of ischemia reperfusion improved renal function, structure and apoptosis through beta common receptor/erythropoietin receptor and PI3K/Akt pathway in a murine model. Exp Biol Med (Maywood).

[15] Yang C, Jia Y, Zhao T, Xue Y, Zhao Z, Zhang J, Wang J, Wang X, Qiu Y, Lin M (2013). Naked caspase 3 small interfering RNA is effective in cold preservation but not in autotransplantation of porcine kidneys. J Surg Res.

[16] Yang C, Li L, Xue Y, Zhao Z, Zhao T, Jia Y, Rong R, Xu M, Nicholson ML, Zhu T, Yang B (2013). Innate immunity activation involved in unprotected porcine auto-transplant kidneys preserved by naked caspase-3 siRNA. J Transl Med.

[17] Yang C, Zhao T, Zhao Z, Jia Y, Li L, Zhang Y, Song M, Rong R, Xu M, Nicholson ML (2014). Serum-stabilized naked caspase-3 siRNA protects autotransplant kidneys in a porcine model. Mol Ther.

[18] Meng XM, Tang PM, Li J, Lan HY (2015). TGF-beta/Smad signaling in renal fibrosis. Front Physiol.

[19] Eddy AA, Neilson EG (2006). Chronic kidney disease progression. J Am Soc Nephrol.

[20] Carew RM, Wang B, Kantharidis P (2012). The role of EMT in renal fibrosis. Cell Tissue Res.

[21] Hers I, Vincent EE, Tavare JM (2011). Akt signalling in health and disease. Cell Signal.

[22] Winbanks CE, Grimwood L, Gasser A, Darby IA, Hewitson TD, Becker GJ (2007). Role of the phosphatidylinositol 3-kinase and mTOR pathways in the regulation of renal fibroblast function and differentiation. Int J Biochem Cell Biol.

[23] Liang Y, Jing Z, Deng H, Li Z, Zhuang Z, Wang S, Wang Y (2015). Soluble epoxide hydrolase inhibition ameliorates proteinuria-induced epithelial-mesenchymal transition by regulating the PI3K-Akt-GSK-3beta signaling pathway. Biochem Biophys Res Commun.

[24] Zhou LN, Wang N, Dong Y, Zhang Y, Zou H, Li Q, Shi Y, Chen L, Zhou W, Han C, Wang Y. Increased Expression of p-Akt correlates with Chronic Allograft Nephropathy in a Rat Kidney Model. Cell Biochem Biophys. 2014. 10.1007/s12013-014-0391-9 25388849

[25] van Rijt WG, Nieuwenhuijs-Moeke GJ, van Goor H, Jespersen B, Ottens PJ, Ploeg RJ, Leuvenink HG (2013). ARA290, a non-erythropoietic EPO derivative, attenuates renal ischemia/reperfusion injury. J Transl Med.

