# Supplementary material for: Cyclic helix B peptide inhibits ischemia reperfusion-induced renal fibrosis via the PI3K/Akt/FoxO3a pathway
Source: J Transl Med. 2015 Nov 10;13:355. doi: 10.1186/s12967-015-0699-2 (PMC4641348; doi:10.1186/s12967-015-0699-2)

**Figure S1**: Validation of FoxO3a siRNA and Wortmannin. The p-FoxO3a proteins (A) and mRNA (B) expression of HK-2 cells were significantly down-regulated by FoxO3a shRNA treatment. The expression of p-Akt was also significantly reduced by Wortmannin in the HK-2 cells (C).


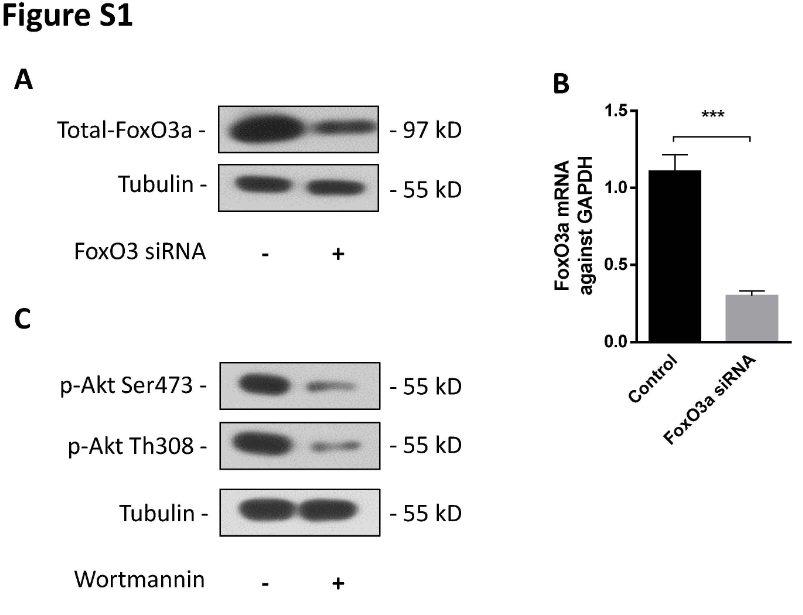

Supplement: Supplementary file 1 — 10.1186/s12967-015-0699-2 Validation of FoxO3a siRNA and Wortmannin. The p-FoxO3a proteins (A) and mRNA (B) expression of HK-2 cells were significantly down-regulated by FoxO3a shRNA treatment. The expression of p-Akt was also significantly reduced by Wortmannin in the HK-2 cells (C). [file 12967_2015_699_MOESM1_ESM.doc]
